# Association of OQLQ Scores with Treatment Acceptance in Orthognathic Surgery: A Cross-Sectional Study

**DOI:** 10.3390/jcm15093428

**Published:** 2026-04-30

**Authors:** Laszlo Seres, Marietta Mucsi, Gellert Perenyi, Andras Kocsis, Robert Paczona

**Affiliations:** 1 Department of Oral and Maxillofacial Surgery, University of Szeged, Kalvaria sgt. 57, 6725 Szeged, Hungarypaczona.robert@med.u-szeged.hu (R.P.); 2 Dr. Andras Kocsis Orthodontic Centre, Marostoi u. 29/A, 6726 Szeged, Hungary; kocsa@fogszabalyzas.hu; 3 Institute of Mathematics, École Polytechnique Fédérale de Lausanne, Rte Cantonale, 1015 Lausanne, Switzerland

**Keywords:** orthognathic surgery, quality of life, patient-reported outcome measures, decision making

## Abstract

**Background/Objectives:** This study aimed to evaluate whether patients’ decisions to undergo orthodontic–surgical treatment for dentofacial deformity are associated with Orthognathic Quality of Life Questionnaire (OQLQ) scores, and to identify which domains show the strongest relationship with treatment acceptance. **Methods:** Adult patients (≥18 years) who were offered orthognathic surgery by the same orthodontic specialist team completed the Hungarian version of the OQLQ. The primary outcome was whether the patient proceeded with surgery following consultation (yes/no). The questionnaire comprises 22 items across four domains, from which domain-specific and total scores (range: 0–88) were calculated. Given the non-normal distribution and group imbalance, comparisons between groups were performed using nonparametric tests, and associations were further evaluated using Firth penalized logistic regression adjusted for age and sex. **Results:** A total of 133 patients were included, of whom 107 proceeded with surgery and 26 did not proceed. Median total OQLQ scores were higher in the surgery group than in the decline group (40.0 [IQR: 27.0–52.0] vs. 31.5 [16.0–52.5]), although this difference was not statistically significant. In adjusted analyses, the total OQLQ score was not independently associated with treatment acceptance. Among the domains, only facial aesthetics showed a modest association that reached statistical significance (OR per 1-point increase: 1.10; 95% CI: 1.002–1.22). **Conclusions:** In this single-center cross-sectional study, OQLQ scores showed only a limited association with treatment acceptance. The observed effect was modest, and only the facial aesthetics domain demonstrated a statistically significant relationship, with a confidence interval close to unity, indicating a borderline and potentially unstable association. These findings suggest that patient-reported quality of life alone is insufficient to explain treatment decisions. The OQLQ may be useful as a supportive tool in clinical consultation, particularly for identifying concerns related to facial appearance, but should not be used as a standalone predictor of whether patients proceed with surgery.

## 1. Introduction

Facial appearance has a substantial impact on individuals’ lives. While a harmonious and attractive facial form may enhance self-esteem, dentofacial deformities can lead to functional, aesthetic, and psychosocial problems, including reduced self-esteem and confidence [1,2,3].

Orthognathic treatment aims not only to achieve long-term occlusal stability but also to improve facial aesthetics and airway function, thereby enhancing the quality of life of patients with dentofacial deformities [4].

Orthognathic treatment is prolonged and requires considerable patient commitment. The decision to undergo surgery represents a complex process that extends beyond clinical indications alone. Factors influencing this decision are not yet fully understood [5].

Previous studies indicate that a large proportion of eligible patients decline surgery [6,7]. Patients are required to weigh the potential functional and aesthetic benefits against the risks, treatment burden, and personal implications of surgery. This process often involves subjective evaluation of expected outcomes, individual tolerance for surgical intervention, and the perceived impact of dentofacial deformity on daily life. As a result, treatment acceptance cannot be fully explained by objective clinical parameters alone. Importantly, the decision to undergo orthognathic surgery is shaped by multiple psychological and contextual factors, and therefore, patient-reported measures such as the OQLQ capture only one component of a complex decision-making process.

Patients’ decisions are influenced by a broad range of factors, including psychological characteristics, expectations, social environment, and access to healthcare resources [5,6]. In particular, perceptions of facial aesthetics and self-image appear to play a central role, as they are closely linked to self-esteem and social functioning [1,2]. At the same time, concerns related to surgical risks, postoperative recovery, and uncertainty regarding outcomes may act as significant barriers to treatment acceptance [6].

Recent patient-centered care models emphasize that treatment decisions arise from shared decision-making processes that integrate clinical evidence with patient preferences, values, and contextual factors, rather than relying on clinical indicators alone [7,8,9].

Effective bidirectional communication between patients and healthcare professionals is essential. The orthognathic team must understand patients’ concerns and expectations, which are critical for treatment planning, while patients must be fully informed about treatment procedures, potential outcomes, benefits, and risks.

However, communication may be hindered by factors such as low self-esteem, reduced self-confidence, and limited consultation time. Oral health-related quality of life questionnaires capture patients’ perceptions of their condition and may facilitate doctor–patient communication.

The Orthognathic Quality of Life Questionnaire (OQLQ) was developed to assess patients with severe dentofacial deformities [10,11]. It comprises 22 items scored on a scale from 0 to 4 (Table 1), yielding a total score ranging from 0 to 88, with higher scores indicating poorer quality of life. The OQLQ includes four domains: (1) oral function (questions 2–6; score 0–20), (2) facial aesthetics (questions 1, 7, 10, 11, and 14; score 0–20), (3) awareness of facial deformity (questions 8, 9, 12, and 13; score 0–16), and (4) social aspects of the deformity (questions 15–22; score 0–32).

Since its introduction in 2000, the OQLQ has been validated in multiple languages, including German, Brazilian Portuguese, Arabic, Spanish, Chinese, Dutch and Hungarian [12,13,14,15,16,17,18,19].

It remains unclear to what extent patient-reported measures are associated with actual treatment decisions. While quality of life instruments, such as the OQLQ, are widely used to assess treatment outcomes, their role in predicting or explaining willingness to undergo surgery has not been well established. A better understanding of this relationship may help improve patient selection, enhance communication, and support shared decision-making in clinical practice.

In addition to facilitating communication, patient-reported outcome measures may also contribute to more individualized treatment planning by helping clinicians better understand the relative importance of functional versus aesthetic concerns from the patient’s perspective. This may be particularly relevant in borderline cases, where the indication for surgery is not solely determined by objective clinical findings but also by patient preferences and expectations. In such situations, structured assessment of patient-reported quality of life may support more transparent and patient-centered decision-making.

In this study, we hypothesized that patients with poorer orthognathic-related quality of life are more likely to accept orthognathic treatment. Therefore, this study aimed to determine whether the OQLQ can differentiate between patients who accept and those who decline orthognathic surgery. Additionally, we sought to identify which items and domains of the OQLQ are most strongly associated with the decision to undergo surgery.

## 2. Methods

In this cross-sectional study conducted between May 2021 and October 2025 at the Dr. Andras Kocsis Orthodontic Centre, Szeged, Hungary, adult patients were consecutively recruited and asked to complete the Hungarian version of the OQLQ. Patients were considered eligible for inclusion after comprehensive orthodontic and surgical assessment, during which the treating team determined that orthognathic surgery was indicated based on skeletal discrepancy, functional impairment, and/or aesthetic considerations. All patients provided written informed consent. Participants were given a 30-min time slot and a private room to complete the questionnaire. The indication for orthognathic surgery had been established prior to questionnaire administration by the orthodontic team.

All participants received the same standardized instructions for questionnaire completion. In addition, the surgeons were guided regarding the information communicated during the consultation. OQLQ analyses were performed on complete questionnaires; one participant with a missing OQLQ response was excluded. Given the minimal extent of missing data, no imputation was performed.

The questionnaire was completed prior to the detailed preoperative consultation with the maxillofacial surgeon. Patients completed the OQLQ independently; no external assistance was provided, except for clarification of questionnaire items upon request. Completed questionnaires were reviewed for completeness at the time of submission. Data were entered into the study database and checked for accuracy to minimize transcription errors. This standardized procedure ensured that OQLQ responses reflected patients’ baseline perceptions and were not influenced by detailed discussion of surgical risks, benefits, or alternatives.

Baseline data, including age and sex, were collected. Following this, the same experienced oral and maxillofacial surgeon who would later perform the surgery conducted the preoperative consultation, during which the diagnosis, treatment plan, surgical procedures, expected functional and aesthetic outcomes, potential risks and complications, and the anticipated treatment schedule were explained in detail. Emphasis was placed on explaining the differences between outcomes achievable with orthognathic surgery and those achievable with orthodontics alone. Patients were also informed about postoperative recovery and the need for their cooperation throughout the entire treatment period. Immediate family members and partners were also invited to participate in the discussions. If the patient requested it, an additional session was scheduled.

Inclusion criteria were age ≥ 18 years, native-level proficiency in Hungarian, and completion of the OQLQ. Exclusion criteria included previous orthognathic surgery and the presence of cleft or other craniofacial disorders. Patients with cleft or other craniofacial disorders were excluded due to their distinct clinical characteristics, treatment pathways, and psychosocial profiles, which could introduce heterogeneity and limit the comparability of OQLQ scores within the study population.

Patients were categorized according to the primary outcome, defined as the patient’s decision, following consultation, to undergo orthognathic surgery (yes/no). The surgery group included patients who had already undergone surgery or were scheduled for surgery, whereas the decline group included patients who did not proceed with surgical treatment. Patients who expressed willingness to undergo treatment but had not yet been scheduled were excluded, as their treatment status was not yet finalized at the time of assessment. Data from the surgery group were compared with those from the decline group.

The primary hypothesis was that higher OQLQ scores (indicating poorer orthognathic-related quality of life) are associated with an increased likelihood of proceeding with orthognathic surgery. Given the relatively small size of the decline group and the ordinal (0–4) response scale of the questionnaire, unadjusted comparisons between groups were performed using the Wilcoxon rank-sum (Mann–Whitney) test [20]. Median scores for the surgery and decline groups are reported, along with two-sided *p*-values.

To account for potential confounding, the association between OQLQ scores and surgical decision was further evaluated using Firth penalized logistic regression models [21,22] to reduce small-sample bias and to obtain stable estimates given the relatively small and imbalanced decline group. Age and sex were included as covariates. Because the total OQLQ score is derived from the sum of correlated domain scores, separate adjusted models were fitted for the total score and for each domain score, rather than including all domains simultaneously, to avoid multicollinearity. Associations are reported as odds ratios per 1-point increase, with 95% confidence intervals.

Item-level analyses were considered exploratory and were undertaken to identify potential response patterns within the questionnaire. However, after adjustment for multiple testing using the Benjamini–Hochberg procedure [23] to control the false discovery rate, no statistically significant associations were identified.

In addition, to support interpretation of the OQLQ as a predictive tool, we assessed the models using the area under the curve (AUC) as a measure of discrimination.

All analyses were performed using R version 4.4.1 [21].

## 3. Results

A total of 153 patients completed the questionnaire. Six patients were excluded (two became pregnant, one postponed surgery due to an upcoming wedding, one moved abroad, and two were lost to follow-up). Data from 14 patients who had initiated presurgical orthodontics but were not yet scheduled for surgery were also excluded, as their final treatment decisions remained uncertain. Consequently, 133 patients were included in the analysis.

Of these, 107 patients underwent or were scheduled to undergo orthognathic surgery (surgery group), while 26 declined surgery following consultation (decline group). Overall, 80.5% of patients (107/133) accepted surgery. Patients in the decline group were older on average, and the proportion of men was lower. Detailed demographic characteristics are presented in Table 2.

Median OQLQ item scores were similar or higher in the surgery group for all items except items 15 and 17. Likewise, median domain-specific and total OQLQ scores were generally higher in the surgery group, with the exception of Domain 4. However, at the 0.05 significance level, only Question 8 showed a statistically significant difference between groups, and no domain-level differences reached statistical significance. After adjustment for multiple testing, the difference for Question 8 was no longer statistically significant. Detailed results are presented in Table 3 and Table 4.

In Firth penalized logistic regression models adjusted for age and sex, the total OQLQ score was not independently associated with surgery acceptance. Among the domains, only the facial aesthetics domain showed a statistically significant positive association, indicating that higher scores (reflecting poorer quality of life) were associated with an increased likelihood of undergoing surgery. This adjusted result should not be interpreted as requiring a statistically significant unadjusted between-group difference at the domain level, because the Wilcoxon analyses and the regression models address different questions: the former compares crude score distributions between groups, whereas the latter estimates the conditional association between domain score and treatment acceptance after accounting for age and sex. No other domains were significantly associated with acceptance. Increasing age was associated with a lower likelihood of proceeding with surgery, whereas male sex was associated with a higher likelihood. Model discrimination was modest overall, with the facial aesthetics domain showing the highest AUC among the domain-specific models; detailed regression results are presented in Table 5, and the model-performance measure is presented in Table 6.

## 4. Discussion

Skeletal facial deformities can negatively affect social functioning and overall well-being [2]. Orthognathic surgery aims to improve facial aesthetics and orofacial function, thereby enhancing quality of life. Although the indications and technical aspects of treatment are well established, the psychosocial determinants of patients’ decision-making remain insufficiently understood. Treatment planning based solely on cephalometric parameters may lead to suboptimal aesthetic outcomes and patient dissatisfaction [24]. While advances in three-dimensional virtual planning have improved outcome predictability, the assessment of facial aesthetics remains largely clinician-driven and may not fully reflect patient priorities [25].

Despite the growing body of literature on objective outcomes of orthognathic treatment, relatively few studies have focused on patient-centered perspectives. Understanding patients’ expectations and concerns is essential for optimizing satisfaction and treatment adherence [26]. Importantly, these expectations may diverge substantially from those of the orthodontic–surgical team and should therefore be systematically explored prior to treatment [27].

Patient-reported outcome measures, such as oral health-related quality of life instruments, provide valuable insight into patients’ subjective experiences. The Oral Health Impact Profile (OHIP) and its shortened version (OHIP-14) are among the most widely used questionnaires for general oral health assessment [28,29].

The Orthognathic Quality of Life Questionnaire (OQLQ) has become a widely accepted tool for assessing the impact of dentofacial deformities. Its widespread validation has enabled more comprehensive evaluation of the relationship between objective clinical findings and subjective patient perceptions.

Several studies have evaluated patient satisfaction following orthognathic treatment [30,31,32,33]. In a recent review, Schaefer et al. identified 23 studies, all of which reported significant improvements in OQLQ scores from pre- to post-treatment, with the greatest changes observed in facial aesthetics and psychological discomfort [34].

Despite generally favorable outcomes, some patients decline the proposed treatment. The decision-making process for orthognathic surgery is complex and multifactorial. Both medical and nonmedical factors have been identified as influencing patients’ decisions [13]. Medical conditions, such as temporomandibular joint disorders and airway-related problems, are not captured by the OQLQ [35,36]. In addition, factors such as fear of pain, surgery, or general anesthesia, concerns about changes in facial appearance, trust in healthcare providers, family and social support, financial constraints, time limitations, and distance to the treatment center may all influence decision-making.

Jawaid et al. reported that the most common reasons for refusing orthognathic surgery were cost and fear of postoperative pain [37]. Similarly, Hågensli et al. found that concerns about potential side effects—particularly nerve injury—treatment burden, and general reluctance to undergo surgery were the most frequently reported reasons for refusal [6].

The present study aimed to investigate the association between OQLQ scores and patients’ decisions to accept or decline orthognathic surgery. A better understanding of the factors underlying refusal, as well as the concerns that most strongly influence decision-making, may help clinicians tailor communication and address patients’ expectations and concerns more effectively.

All patients included in this study were diagnosed with severe skeletal malocclusions and functional disorders and were therefore offered orthognathic surgery. In our sample, patients who accepted surgery tended to report poorer orthognathic-related quality of life (i.e., higher OQLQ scores) than those who declined. However, these differences were generally small and did not reach statistical significance at the 0.05 level, and the overall OQLQ total score was not independently associated with acceptance after adjustment for key covariates. Among the four domains, facial aesthetics showed the strongest and only statistically significant association with treatment acceptance. However, these findings should be interpreted with caution given the substantial imbalance between groups, particularly the relatively small size of the decline group. Although Firth penalized logistic regression was used to reduce small-sample bias, the limited number of events restricts the stability and precision of the estimates. This is reflected in the width of the confidence intervals and the borderline nature of the observed association for the facial aesthetics domain. Consequently, these results should be considered exploratory, and the observed associations should not be interpreted as robust or directly actionable in clinical decision-making.

These findings suggest that patient-reported quality of life alone may not fully explain treatment decisions. The relatively weak association observed between OQLQ scores and treatment acceptance likely reflects the multifactorial nature of decision-making in orthognathic patients. While patient-reported outcome measures capture the subjective burden of dentofacial deformity, they do not fully account for individual differences in risk tolerance, coping strategies, or attitudes toward surgical intervention. For instance, two patients with similar OQLQ scores may differ substantially in their willingness to undergo surgery due to differences in fear of complications, perceived treatment burden, or personal priorities. This perspective is consistent with contemporary frameworks of shared decision-making, which describe treatment choices as the result of interactions between clinical information, individual values, risk perceptions, and situational constraints [7,8,9]. In this context, patient-reported outcome measures such as the OQLQ provide insight into perceived burden but represent only one component of a broader decision-making process.

In addition, external factors such as financial considerations, time constraints, and social support may further influence decision-making independently of perceived quality of life. This may explain why some patients with relatively high OQLQ scores decline treatment, while others with lower scores proceed with surgery. These factors were not directly assessed in the present study and are provided to contextualize the findings rather than to explain the observed associations.

These findings may indicate that concerns related to facial appearance play a role in decision-making; however, this observation should be interpreted cautiously given the potential for residual confounding and the limited strength of the association. Orthodontic practice often places greater emphasis on clinician-driven outcome measures than on patient-reported outcomes [38,39]. Our findings underscore the importance of exploring patients’ expectations regarding facial aesthetics in greater depth during consultations and incorporating these considerations into treatment planning.

From a clinical perspective, these findings emphasize the importance of integrating patient-reported outcomes into routine orthognathic consultation workflows. The OQLQ may help clinicians identify discrepancies between objective clinical indications and subjective patient concerns, particularly in relation to facial aesthetics.

Furthermore, the use of the OQLQ may assist in identifying patients who require additional counseling or psychological support prior to making a treatment decision. For example, high scores in domains related to self-consciousness or social discomfort may indicate increased vulnerability and the need for more detailed discussion regarding expected outcomes and potential changes in appearance.

The OQLQ may therefore serve as a useful adjunct in clinical consultations by helping to identify patient-reported concerns related to facial aesthetics, oral function, and psychosocial impact. It can facilitate structured communication, highlight domains of particular importance to the patient, and support shared decision-making. Elevated scores in the facial aesthetics domain may indicate areas that warrant more detailed discussion during treatment planning.

However, clinicians should not rely on OQLQ scores as a standalone tool for predicting willingness to undergo orthognathic surgery; see Table 6 for the modest discriminative performance of the adjusted models. The questionnaire does not capture important determinants of decision-making, such as psychological factors (e.g., anxiety, risk perception), socioeconomic constraints, or individual preferences. Therefore, OQLQ results should be interpreted within the broader clinical and psychosocial context of each patient.

## 5. Limitations

This study has several limitations that should be considered when interpreting the findings. Its single-center design may limit generalizability. The study population represents a preselected group of patients already deemed suitable candidates for orthognathic surgery, which limits the generalizability of the findings to broader patient populations with dentofacial deformities. Group classification may have been influenced not only by patients’ willingness but also by logistical and treatment-related factors, which may limit the extent to which the outcome reflects pure decision-making. The relatively small size of the decline group results in an imbalance between the comparison groups, reducing statistical power despite the use of bias-reducing methods. Due to the cross-sectional design, causal relationships cannot be established. In addition, as a self-reported measure, the OQLQ is subject to response bias and individual interpretation, and may not capture all relevant psychological and socioeconomic factors influencing decision-making. Patients’ perceptions may therefore have been influenced by factors not captured in the study. Finally, selection bias cannot be excluded, as only patients with a definitive treatment decision were included.

## 6. Conclusions

The Orthognathic Quality of Life Questionnaire (OQLQ) is a useful tool for assessing patients’ orthognathic-related quality of life and for identifying concerns related to facial appearance and function that may inform clinical consultations, although its impact on communication or decision-making was not directly assessed in this study. Incorporating patient-reported concerns into the decision-making process may improve communication and support shared decision-making.

In this analysis, OQLQ scores alone were not sufficient to reliably distinguish between patients who accepted and those who declined surgery, and the evidence for an independent association was limited, particularly due to the relatively small size of the decline group.

Among the OQLQ domains, facial aesthetics showed the strongest observed association after adjustment for age and sex. This finding should be interpreted cautiously, because treatment acceptance is multifactorial and residual confounding from unmeasured determinants is likely. Even so, the comparative prominence of the facial aesthetics domain suggests that appearance-related concerns may be particularly relevant during patient consultation.

Future multicenter and longitudinal studies with larger and more balanced samples are warranted to validate these findings and to better characterize the complex factors underlying patient decision-making.

## Figures and Tables

**Table 1:** Orthognathic Quality of Life Questionnaire.

	Domain	Questions
1	2	I am self-conscious about the appearance of my teeth.
2	1	I have problems biting.
3	1	I have problems chewing.
4	1	There are some foods I avoid eating because the way my teeth meet makes it difficult
5	1	I don’t like eating in public places.
6	1	I get pains in my face or jaw.
7	2	I don’t like seeing a side view of my face (profile).
8	3	I spend a lot of time studying my face in the mirror.
9	3	I spend a lot of time studying my teeth in the mirror.
10	2	I dislike having my photograph taken.
11	2	I dislike being seen on video.
12	3	I often stare at other people’s teeth.
13	3	I often stare at other people’s faces.
14	2	I am self-conscious about my facial appearance.
15	4	I try to cover my mouth when I meet people for the first time.
16	4	I worry about meeting people for the first time.
17	4	I worry that people will make hurtful comments about my appearance.
18	4	I lack confidence when I am out socially.
19	4	I do not like smiling when I meet people.
20	4	I sometimes get depressed about my appearance.
21	4	I sometimes think that people are staring at me.
22	4	Comments about my appearance truly upset me, even when I know people are only joking.

**Table 2:** Participant characteristics by decision group.

Group	n	Age Mean (SD)	Male n (%)
Accepted surgery	107	27.7 (7.5)	53 (49.5)
Declined surgery	26	32.9 (10.7)	9 (34.6)

**Table 3:** Median OQLQ scores by decision group, and the *p*-value of the Wilcoxon test.

Domain	Accepted (n = 107)	Declined (n = 26)	*p*-Value (Wilcoxon Rank-Sum Test)
OQLQ total, 0–88 (median [IQR])	40.0 (27.0–52.0)	31.5 (16.0–52.5)	0.252
Domain 1: oral function, 0–20 (median [IQR])	8.0 (3.0–12.0)	5.0 (3.25–10.0)	0.389
Domain 2: facial aesthetics, 0–20 (median [IQR])	14.0 (11.0–17.5)	12.0 (7.25–16.0)	0.132
Domain 3: awareness, 0–16 (median [IQR])	9.0 (5.0–11.0)	7.0 (1.0–11.5)	0.100
Domain 4: social aspects, 0–32 (median [IQR])	9.0 (3.0–17.5)	9.5 (2.25–16.75)	0.898

**Table 4:** OQLQ item-level comparisons by decision group.

Question	Domain	Accepted Median (IQR)	Declined Median (IQR)	*p*-Value	q-Value (Benjamini–Hochberg Adjusted)
Q1	Facial aesthetics	4 (3–4)	4 (2–4)	0.714	0.872
Q2	Oral function	3 (1–4)	3 (1–3.75)	0.674	0.872
Q3	Oral function	2 (1–3)	1.5 (1–2.75)	0.209	0.747
Q4	Oral function	1 (0–2)	1 (0–2)	0.867	0.953
Q5	Oral function	0 (0–2)	0 (0–2)	0.805	0.932
Q6	Oral function	0 (0–3)	0 (0–1)	0.238	0.747
Q7	Facial aesthetics	4 (2.5–4)	3 (1–4)	0.065	0.480
Q8	Awareness of deformity	2 (1–3)	0.5 (0–3)	0.039	0.480
Q9	Awareness of deformity	2 (1–3)	1 (0–3)	0.066	0.480
Q10	Facial aesthetics	3 (1.5–4)	2 (1–4)	0.367	0.782
Q11	Facial aesthetics	3 (1–4)	2 (1–4)	0.532	0.782
Q12	Awareness of deformity	2 (1–3)	2 (0–3)	0.514	0.782
Q13	Awareness of deformity	2 (1–3)	1.5 (0–4)	0.318	0.777
Q14	Facial aesthetics	3 (2–4)	2 (0.25–4)	0.207	0.747
Q15	Social aspects	0 (0–2)	1 (0–3)	0.185	0.747
Q16	Social aspects	0 (0–2)	0 (0–2)	0.965	0.965
Q17	Social aspects	0 (0–2)	1 (0–2)	0.408	0.782
Q18	Social aspects	1 (0–3)	1 (0.25–2)	0.650	0.872
Q19	Social aspects	2 (0–3)	1 (0–3)	0.512	0.782
Q20	Social aspects	2 (0–3)	1 (0–3)	0.533	0.782
Q21	Social aspects	1 (0–2)	0 (0–2)	0.318	0.777
Q22	Social aspects	1 (0–2)	1 (0–2.75)	0.953	0.965

**Table 5:** Associations with willingness to proceed, adjusted for age and sex (Firth logistic regression).

Model	OQLQ OR (95% CI)	OQLQ (*p*)	Age OR (95% CI)	Sex = Man OR (95% CI)
Total	1.02 (0.99–1.05)	0.151	0.94 (0.90–0.99)	2.22 (0.85–6.10)
Domain 1: Oral	1.08 (0.99–1.18)	0.089	0.93 (0.89–0.98)	2.17 (0.86–5.76)
Domain 2: Facial	1.11 (1.01–1.22)	0.031	0.94 (0.89–0.99)	2.48 (0.95–7.03)
Domain 3: Awareness	1.09 (0.98–1.21)	0.106	0.95 (0.90–0.99)	2.15 (0.85–5.71)
Domain 4: Social	0.99 (0.95–1.05)	0.838	0.94 (0.89–0.98)	1.68 (0.68–4.32)

**Table 6:** Predictive performance of the adjusted models.

Model	Area Under the Curve (AUC)
Total	0.708
Domain 1: Oral	0.706
Domain 2: Facial	0.719
Domain 3: Awareness	0.686
Domain 4: Social	0.689

## Data Availability

The raw data supporting the conclusions of this article will be made available by the authors on request.
